# Supplementary material for: ATPase Inhibitory Factor-1 Disrupts Mitochondrial Ca2+ Handling and Promotes Pathological Cardiac Hypertrophy through CaMKIIδ
Source: Int J Mol Sci. 2021 Apr 23;22(9):4427. doi: 10.3390/ijms22094427 (PMC8122940; doi:10.3390/ijms22094427)
Supplement: Supplementary file 1 [file ijms-22-04427-s001.zip › ijms-1164691-supplementary.pdf]

Supplemental material

Supplemental table 1. Antibodies used for immunoblotting

Antibody	Company	Catalog number
ATPIF1 (human)	SCBT	sc-271614
ATPIF1 (total)	Invitrogen	A-21355
GAPDH	Fitzgerald	10r-g109a
CAMKII (Thr286)	Cell signaling	12716
CAMKII (PAN)	Cell signaling	4436S
AMPK (thr172)	Cell signaling	2535
AMPK	Cell signaling	2532
PLN (ser16)	Badrilla	A010-12AP
PLN (thr17)	Badrilla	A010-13AP
PLN	ABCAM	ab126174
PINK1	Cell signaling	6946
PARKIN	Cell signaling	4211
DRP1	Cell signaling	5391
COX4	Cell signaling	4850
mtHSP70	ThermoFisher	MA3-028
OXPHOS	ABCAM	ab110413
α-actinin	ABCAM	ab72592
MFN2	Cell signaling	11925

Supplemental table 2. Primers sequences list used for RT-qPCR

Gene		Forward	Reverse
ATP inhibitor factor-1 (IF1)	Mouse	GGAGCCTTCGGAAAACGAGA	ATGGTGTTTCCTCAGGGCAG
ATP inhibitor factor-1 (IF1)	Human	CAGTCCGAGAATGTCGACCG	CAGTTGTTCTCTACTCTGTG
ATP inhibitor factor-1 (IF1)	Rat	GTCGGAGAGCATGGATTCGG	GCCAGCTGCTCTCTAGTCTT
Lactate dehydrogenase (LDH)	Rat	GTGCACTAAGCGGTCCCAAA	TGTTCTGGGGGACCTGTTCT
Pyruvate kinase (PRK)	Rat	GCACCTGATAGCTCGAGAGG	AGGGGGTCTGTGGATTGACT
NAPDH oxidase 2 (NOX2)	Rat	CTCGACAAGGATTCGAAGAC	GTGCTATCATCCAAGCTACC
Nuclear receptor factor (NRF2)	Rat	TTGGAGCACTTACTGGAGTC	CTTCCGCCATAATGAATCCC
Heat shock protein 60 (HSP60)	Rat	TTCCTCAGAGGTTGGCTATG	ATTCCAGGGTCCTTCTCTTC
Atrium natriuretic peptide (ANP)	Rat	ATGGGCTCCTTCTCCATCAC	TCTACCGGCATCTTCTCCTC
Brain natriuretic peptide (BNP)	Rat	ACAATCCACGATGCAGAAGCT	GGGCCTTGGTCCTTTGAGA
Regulator of calcineurin 1 (RCAN1)	Rat	GTCACGGCTGTTACCTCCAA	GCCAGAGTACACACCCATCC
CYP (mtDNA)	Rat	CCTCCCATTCATTATCGCCGCCCTTGC	GTCTGGGTCTCCTAGTAGGTCTGGGAAA
TRPM2 (ncDNA)	Rat	GTACAACGAGCTGCTTCATTCC	GCACCTCTAAGAGGCATCCATC
Short fraction D-Loop	Rat	CCTCCCATTCATTATCGCCGCCCTTGC	GTCTGGGTCTCCTAGTAGGTCTGGGAA
Long fraction D-Loop	Rat	AAAATCCCCGCAAACAATGACCACCC	GGCAATTAAGAGTGGGATGGAGCCAA
36B2	Rat	GTTGCCTCAGTGCCTCACTC	GCAGCCGCAAATGCAGATGG

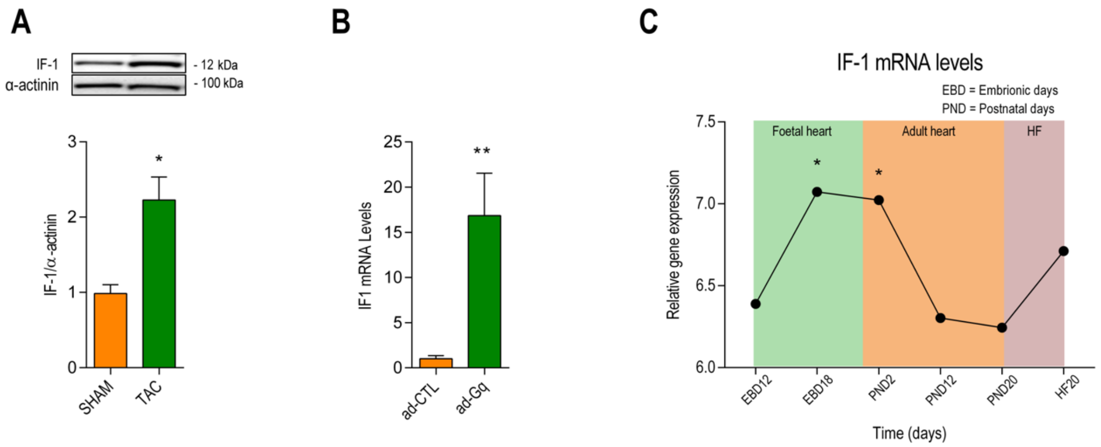

Figure S1. IF1 gene expression during embryonic development and heart failure. (A) IF1 protein level normalized for alpha-actinin detected by western blot in lysates of mice subjected to TAC or sham surgery (n=5). **(B)** Cardiac IF-1 mRNA expression at embryonic day (EBD) 12 and 18, post-natal day 2 and 20. IF1 is expressed at values relative to EBD12. In addition, IF1 expression was compared between 20-week-old mice with heart failure (HF) after myocardial infarction and sham controls (n=3). *p < 0.05 vs EBD12. **(C)** IF-1 mRNA expression in neonatal rat ventricular myocytes (NRVM) infected with an adenovirus expression constitutively activated Gαq (ad-Gq) or a control virus (ad-CTL) for 48 hours (n=5). Data are presented as mean ± SEM. *p < 0.05 and **p < 0.01 vs SHAM/ ad-CTL/EBD12 by nonparametric Mann-Whitney test.

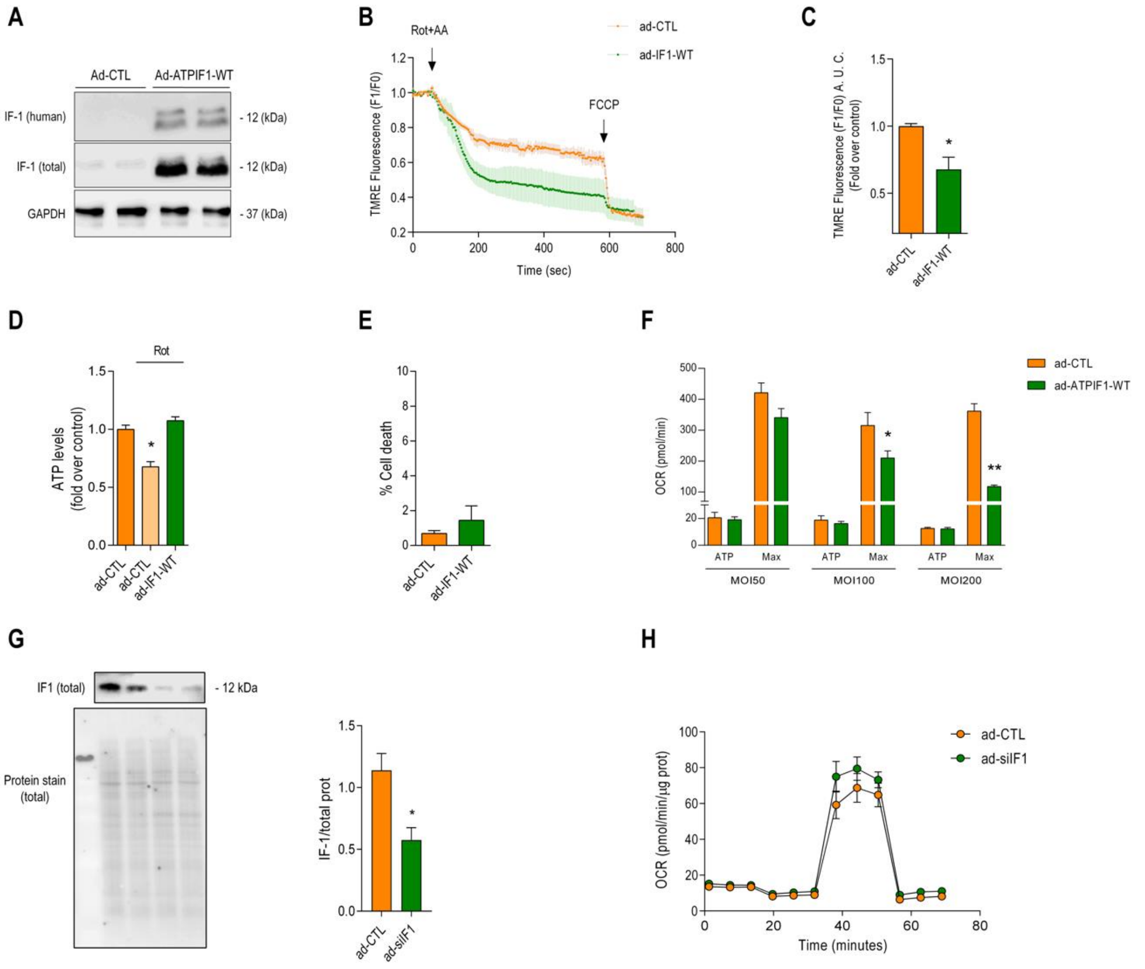

Figure S2. Effect of IF1 on mitochondrial membrane potential, ATP levels and cell death after respiratory collapse. Neonatal rat ventricular myocytes (NRVM) were infected with an adenoviral vector expressing the human IF1 (ad-IF1-WT), or an empty vector control virus (ad-CTL) for 48 hrs. **(A)** Representative western blot image from whole cell lysates using an antibody specific for the human isoform of IF-1 and an antibody that recognises all IF-1 isoforms. Glyceraldehyde-3-Phosphate dehydrogenase (GAPHD) served as loading control. **(B)** Time-lapse of the mitochondrial membrane potential measured with Tetramethylrhodamine, Ethyl Ester, Perchlorate (TMRE) before and after serial addition of rotenone (rot) + Antimycin-A (AA) or FCCP. Graphs represent 5 independent experiment (n=5). **(C)** Bar graph depicting the area under the curve of TMRE fluorescence starting from the addition of rot+aa until the addition of FCCP (n=5). **(D)** Intracellular ATP levels measured in the presence or absence of incubation with rotenone for 24 h. Intracellular ATP levels were detected using microplate colorimetric reader. (n=4). **(E)** ATP-linked and maximal mitochondrial respiration of NRVC infected with different adenovirus concentrations (MOI 50, 100 and 200). **(F)** NRVM were stained with the live cell indicator Calcein® and propidium iodide after infection with ad-IF1-WT and ad-Ctl for 48 hours. A total of 5 different regions of interest were selected per well and the total number of dead cells was expressed as a percentage to the total number of cells. (n=4). **(G)** Representative immunoblot from

whole cell lysate using an antibody specific total IF1 after adenovirus infection that overexpress a small interference against IF1 (siIF1) or scrambled siRNA (ad-CTL, left panel). Protein levels of IF1 normalized to total protein (n=4, right panel). **(H)** Typical SeaHorse experiment depicting oxygen consumption rate (OCR) of NRVM after serial treatments with oligomycin (oligo), FCCP and rot + AA. The graph represents 4 independent experiments. * $p < 0.05$, ** $p < 0.01$ and *** $p < 0.001$ vs ad-CTL using the ad-CTL using the Mann-Whitney U test or T-test where appropriate.

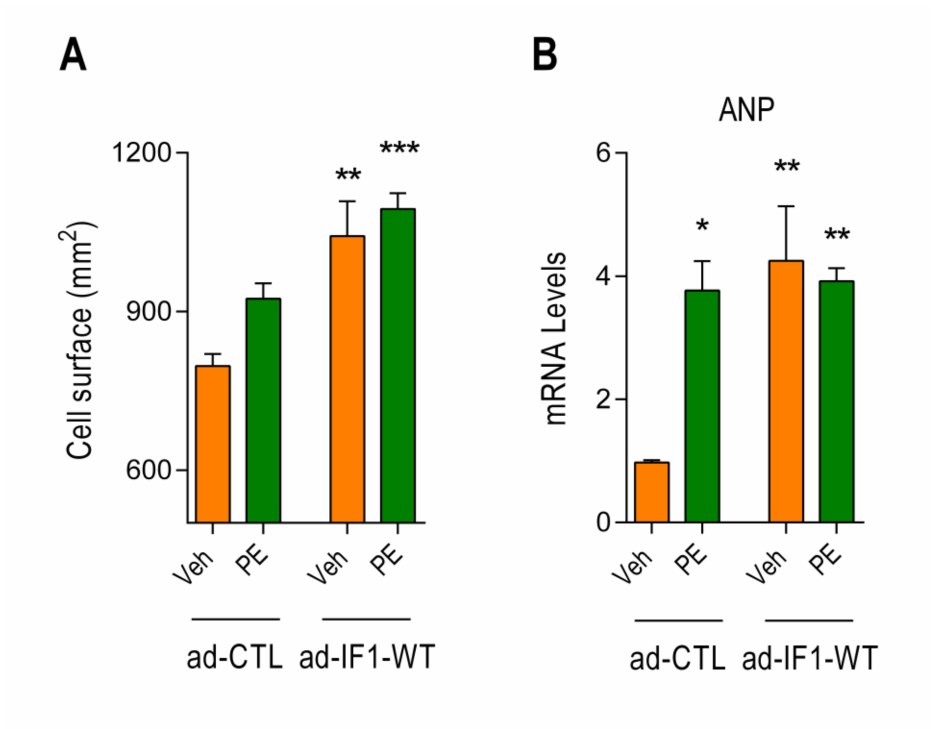

Figure **S3. Effect of IF1 expression on phenylephrine-induced cardiomyocyte hypertrophy.** NRVMs were infected with ad-IF1-E55A or ad-CTL for 48 hrs. **(A)** Bar graph depicting differences in cardiomyocyte size in the presence or absence of phenylephrine (50 μM) (n=4). **(B)** ANP mRNA expression of cells infected as in **A.** (n=4). * $p < 0.05$, ** $p < 0.01$ and *** $p < 0.001$ vs ad-CTL using the ad-CTL using Kruskal Wallis followed by the Mann-Whitney U post-hoc test.

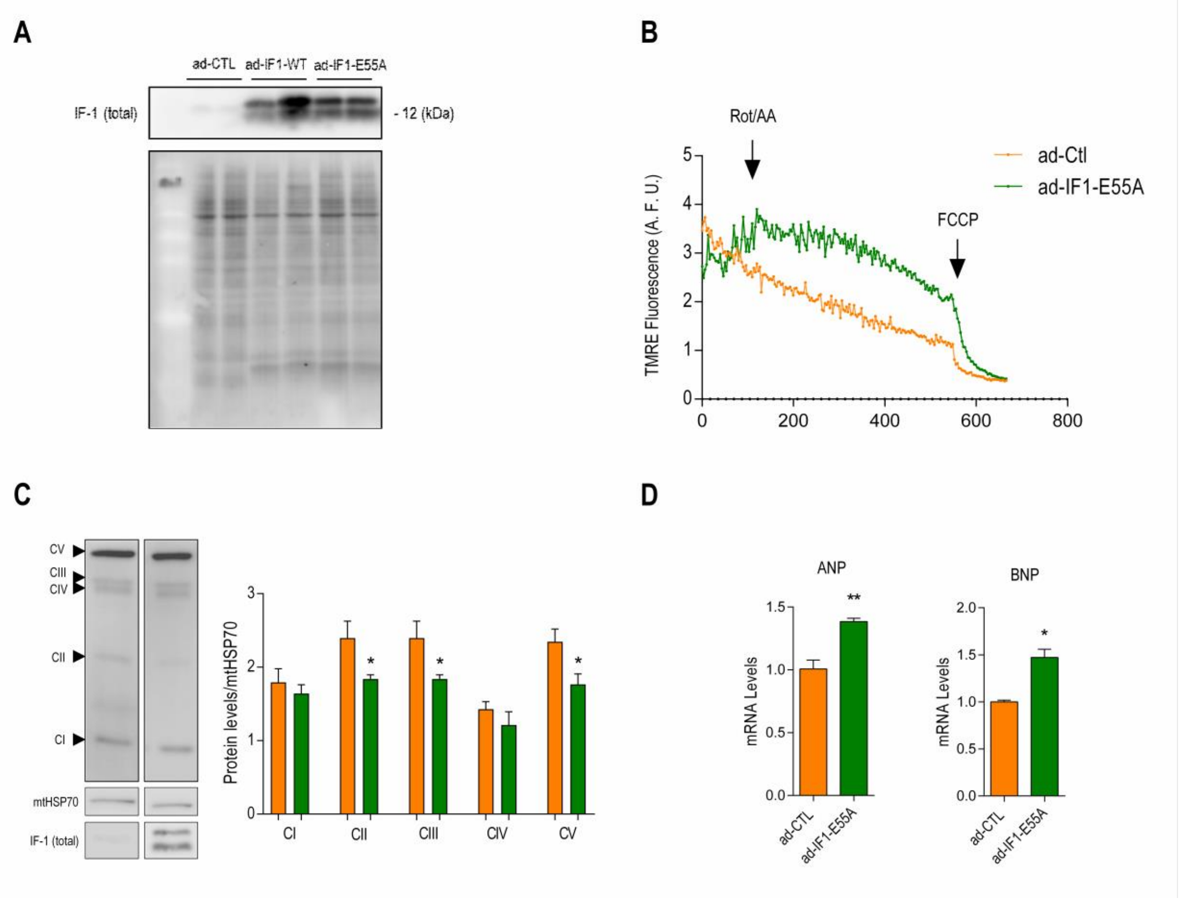

Figure S4. An IF-1 mutant incapable of binding to ATP-synthase still downregulates respiratory chain complexes and stimulates natriuretic peptides. NRVMs were infected with ad-IF1-E55A and ad-CTL for 48 hrs. **(A)** Representative western blot image from whole cell lysate using an antibody that recognises all IF-1 isoforms (top) from cells infected with ad-CTL (left), ad-IF1-WT (middle) and ad-IF1-E55A (right). Total protein stained using Fluorescence REVERT® solution (bottom). **(B)** Representative traces of mitochondrial membrane potential from cells infected with ad-IF1-E55A or ad-CTL measured with TMRE before and after serial addition of rotenone (rot) + Antimycin-A (AA) and FCCP. **(C)** Changes in electron transport chain protein complexes detected with western blot (n=3). **(D)** mRNA expression of the natriuretic peptides ANP and BNP (n=4). * $p < 0.05$, ** $p < 0.01$ and *** $p < 0.001$ vs ad-CTL using the ad-CTL using the Mann-Whitney U test or T-test where appropriate.

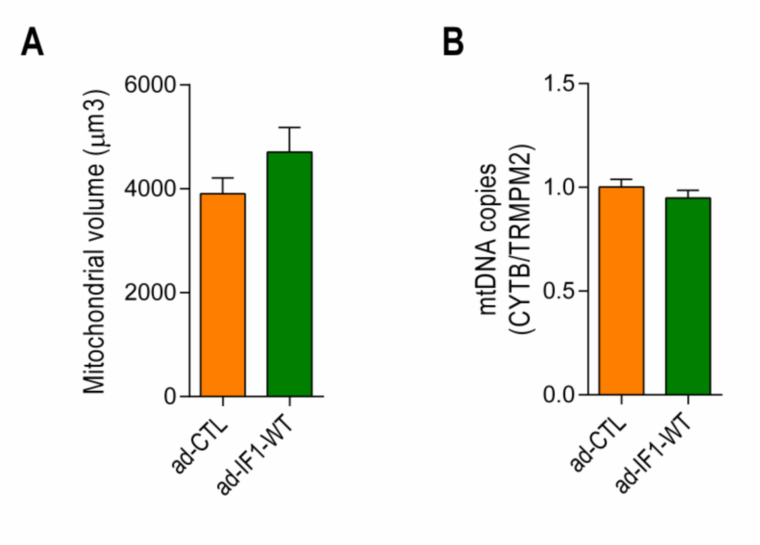

Figure S5. IF1 overexpression does not affect mitochondrial content. NRVMs were infected with ad-IF1-WT or ad-CTL for 48 hrs. **(A)** Bar graph depicting changes in the total mitochondrial volume per cell. (ad-CTL; n=17 cells and ad-IF1-WT; n=18 cells) using confocal microscope as described in Fig 5. **(B)** Bar graph depicting the ratio between mitochondrial DNA (cytochrome B, CYTB) and nuclear DNA (Transient receptor potential cation channel subfamily M member-2, TRPM-2) in cells infected with ad-IF1-WT or ad-CTL (n=4).

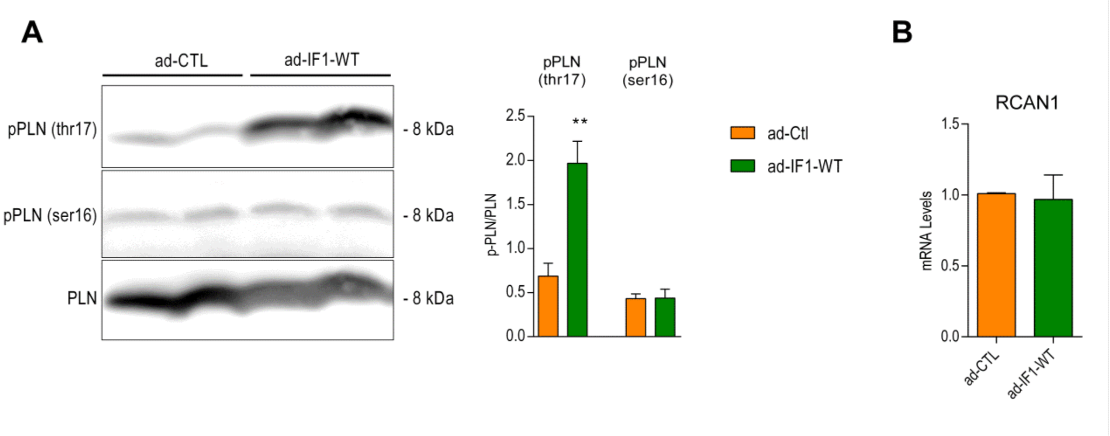

Figure S6. Effects of IF-1 CaMKII-dependent and PKA-dependent phosphorylation of Phospholambam. NRVMs were infected with ad-IF1-WT or ad-CTL for 48 hrs. **(A)** Right panel: Representative immunoblot from whole cell lysate using specifics antibodies to detect total Phospholambam (PLN) as well as PLN phosphorylation at the CaMKII specific (threonine 17) and the protein kinase A specific (serine 16) phosphorylation sites. Left Pannel: Bar graph depicting changes in phospholamban phosphorylation (n=4). **(B)** mRNA levels of the Regulator of calcineurin- 1 (RCAN1) (n=4). **p < 0.01 vs ad-Ctrl* using the Mann-Whitney U test.

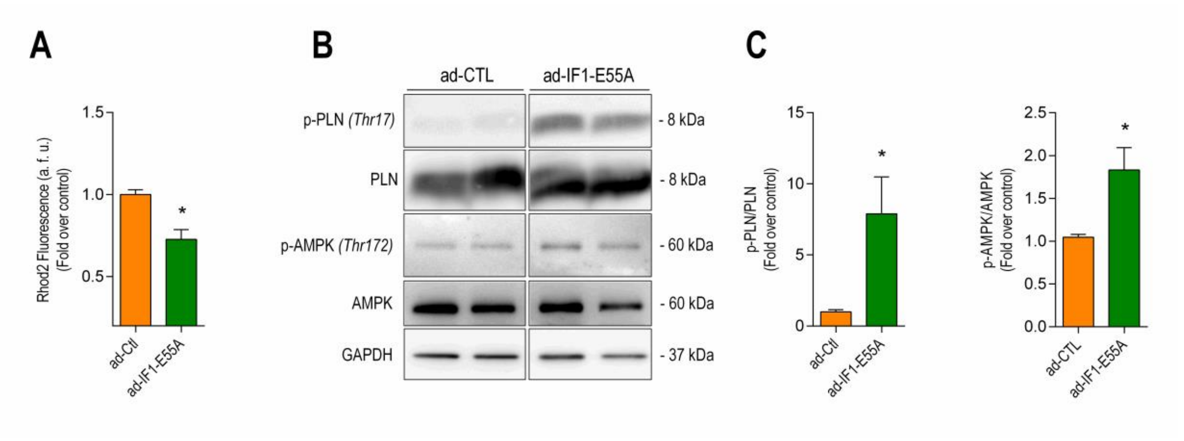

Figure S7. IF-1 mutant sufficient to reduce mitochondrial calcium and activates CAMKII signaling. NRVMs were infected with ad-IF1-E55A and ad-CTL for 48 hrs. **(A)** Bar graph depicting basal mitochondrial Ca^{2+} labelled with Rhod-2 AM. (n=3). **(B and C)** Protein levels of phosphorylated Phospholamban (threonine 17) (n=3) and AMPK (threonine 172) (n=5) were assessed with western blot. *p < 0.05 vs ad-Ctrl using the Mann-Whitney U test.
